# Incidence of De Quervain’s thyroiditis during the COVID-19 pandemic in an area heavily affected by Sars-CoV-2 infection

**DOI:** 10.1007/s12020-021-02841-8

**Published:** 2021-08-07

**Authors:** Ilenia Pirola, Elena Gandossi, Mario Rotondi, Fiorella Marini, Alessandra Cristiano, Luca Chiovato, Maurizio Castellano, Alberto Ferlin, Carlo Cappelli

**Affiliations:** 1 Department of Clinical and Experimental Sciences, SSD Medicina ad Indirizzo Endocrino-Metabolico, ASST Spedali Civili di Brescia, University of Brescia, 25123 Brescia, Italy; 2 Unit of Internal Medicine and Endocrinology, ICS Maugeri I.R.C.C.S., Laboratory for Endocrine Disruptors, University of Pavia, Pavia, Italy

**Keywords:** COVID-19, De Quervain, Thyrotoxicosis, Subacute thyroiditis

## Abstract

**Purpose:**

To evaluate the possible association between Covid-19 infection and subacute thyroiditis.

**Methods:**

We reviewed the medical and imaging records of patients referred to our Department’s outpatient setting dedicated to ‘thyroid emergency’ (records with a ‘*bollino verde*’—green sticker, classifed as urgent) from April 2020 to October 2020. This outpatient clinic is devoted to patients requiring evaluation for severe hypothyroidism, thyrotoxicosis and neck discomfort or pain. All patients with a newly-diagnosed subacute thyroiditis were selected. The data of all patients receiving a diagnosis of subacute thyroiditis was collected retrospectively, taking into account the same period of time (April–October) and starting from 2016.

**Results:**

During the COVID-19 outbreak in our region (April 2020 to October 2020) 396 patients attended the outpatient emergency clinic. Among them, 10 (2.5%) patients received a diagnosis of subacute thyroiditis. In a single patient, a 44-year-old man, a COVID-19 pulmonary infection had been diagnosed 7 weeks before the diagnosis of subacute thyroiditis. All of the remaining patients were and remain COVID-19 free as confirmed by telephone interview. The percentage of patients who received a diagnosis of subacute thyroiditis in the same period starting from 2016 was very similar (2.9%, 2.9%, 2.6% and 3.0% in 2016, 2017, 2018 and 2019, respectively).

**Conclusions:**

Our data do not show an increase in the incidence of subacute thyroiditis in the Brescia area, a region with the highest prevalence of COVID-19 in Italy during the period of the pandemic outbreak.

## Introduction

Among the countries of the European Union, COVID-19 infections spread most drastically in Italy in February 2020. Northern Italy, and in particular the Bergamo and Brescia areas, showed the highest number of patients affected by COVID-19, with a dramatic increase in deaths related to COVID-19 infections between February and April 2020 [1].

Subacute thyroiditis (SAT), or De Quervain’s thyroiditis, was described for the first time in 1904 and is characterised by neck pain or discomfort and thyroid dysfunction [2]. SAT is often caused by a viral infection or a postviral inflammatory process mainly of the upper respiratory tract [3]. In fact, many patients have a history of upper respiratory infection prior to the onset of thyroiditis, mainly 2–8 weeks beforehand. Thyrotoxicosis is the typical presentation followed by euthyroidism, transient hypothyroidism, and ultimately restoration of normal thyroid function. The diagnosis of SAT is largely based on clinical symptoms, but laboratory findings [i.e. high erythrocyte sedimentation rate and/or C-reactive protein level (CRP)], and/or a low radioiodine uptake during the thyrotoxic phase or typical ultrasound features are helpful in confirming the diagnosis [4].

Recently, several reports have described patients affected by COVID-19 infection who developed SAT, suggesting that thyroid tissue may be a possible target of the virus.

*The aim of the present study was to evaluate whether the Sars-CoV-2 pandemic has lead to an increase in the number of patients diagnosed with SAT as compared to pre-pandemic levels. To this end, the number of patients who received a diagnosis of SAT during the pandemic outbreak (from April 2020 to October 2020) was compared to the number of patients diagnosed with SAT during the same time period (April–October) of the previous 4 years (2016–2019)*.

## Subjects and methods

We reviewed the medical and imaging records of patients referred to our Department’s outpatient setting dedicated to ‘thyroid emergency’ (records with a ‘*bollino verde’*—green sticker, classifed as urgent) from April 2020 to October 2020. This outpatient clinic is devoted to patients requiring evaluation for severe hypothyroidism, thyrotoxicosis and neck discomfort or pain. All patients with newly-diagnosed SAT were selected. The diagnosis was mainly clinical and supported by elevation of CRP and/or EAS levels, thyrotoxicosis and at least one instrumental image of either low radioiodine uptake at scintiscan or typical ultrasound features [4].

Serum concentrations of TSH (normal range 0.4 ± 4.5 mIU/L, analytical sensitivity 0.004 mIU/L) and fT4 (normal range 8.0–19.0 pg/mL, analytical sensitivity 1 pg/mL) were measured using a fully automated Architect i2000 analyser (Abbott Diagnostics, Abbott Park, IL, USA) using chemiluminescent magnetic immunoassays. For WBC count, specimens were collected in a peripheral blood sampling microtainer tube containing K_2_EDTA and analysed using an automated blood analyser (Coulter LH 750) within 4 h from collection. To determine hsCRP plasma levels, blood was collected in a lithium-heparin tube and concentrations were measured using an immunoassay technique (Siemens Healthcare Diagnostics, Den Hague, the Netherlands) with a lower detection limit of 0.2 mg/L. The instruments were calibrated against appropriate proprietary reference standard material and verified by using the registered quality controls.

The scintigraphy (technetium-99m pertechnetate) was performed using a gamma camera fitted with a low-energy high-resolution collimator. Multiple views were acquired including anterior, lateral and oblique views.

Ultrasounds were performed by a skilled sonographer with more than 10 years of experience, blind with respect to both TSH values and scinigraphy results and skilled in SAT diagnosis [4]; an AplioTM500 (Toshiba Medical Systems Corp, Otawara, Japan) ultrasonographic scanner was used, fitted with a 10–14 MHz linear transducer for morphological study.

All patients provided written informed consent for the storage and use of their data. The study was approved by the Local Ethical Committee (no. 4631).

### Statistical analysis

All data were collected in an electronic case report database. Comparisons between groups and the difference between proportions were calculated using *χ*^2^ for categorical variables and ANOVA test for quantitative variables, as appropriate. Two-tailed *p* < 0.05 was considered statistically significant. Statistical analyses were performed using SPSS 20.0 software (SPSS, Inc., Evanston, IL, USA).

## Results

During the COVID-19 outbreak in our region, (April 2020 to October 2020), 396 patients attended the ‘bollini verdi’ emergency outpatient clinic. Among them, 10 (2.5%) patients received a diagnosis of SAT. Nine patients showed neck pain and one had arthralgia and tremor spreading to the extremities.

In a single patient, a 44-year-old man, a COVID-19 pulmonary infection had been diagnosed 7 weeks before the diagnosis of SAT; he did not require hospitalisation, and was treated with azithromycin, chloroquine, corticosteroids and low molecular weight heparin by subcutaneous injection at home. All of the remaining patients were and remain COVID-19 free as confirmed by telephone interview.

All the patients were submitted to a thyroid ultrasound that was suggestive of SAT. Three subjects had previously performed technetium-99m pertechnetate scintigraphy that showed absence of or very low radioiodine uptake.

The data of all patients who received a diagnosis of SAT was collected retrospectively, taking into account the same period of time (April–October) starting from 2016.

A diagnosis of SAT was made in 9/310 (2.9%), 11/379 (2.9%), 10/384 (2.6%) and 12/400 (3.0%) patients evaluated at the ‘bollini verdi’ emergency outpatient clinic in 2016, 2017, 2018 and 2019, respectively.

The clinical characteristics of all patients are shown in Table 1.

**Table 1:** Clinical and biochemical characteristics of all patients enrolled in the study

From April 2020 to October of each year						
	2016	2017	2018	2019	2020	*p* value
Gender (f/m)	6/3	8/3	6/4	9/3	7/3	0.954
Age (yrs)	41.6 (3.2)	42.1 (2.6)	41.9 (3.6)	42.2 (3.6)	41.9 (3.1)	0.993
WBC	13 (1.4)	12.6 (1.2)	13.1 (1.5)	12.4 (1.2)	12.8 (1.3)	0.757
CRP (mg/L)	26.2 (5.5)	26.9 (7.4)	33.9 (5.6)	29.4 (5.1)	27.2 (6.6)	0.254
ESR (mm/h)	58.3 (8.7)	58.2 (8.4)	55.4 (7.1)	56.9 (8.9)	55.5 (8.5)	0.884
TSH (mIU/L)	0.08 (0.02)	0.03 (0.03)	0.06 (0.11)	0.06 (0.04)	0.05 (0.04)	0.745
fT4 (pg/mL)	21.3 (1.6)	21.5 (1.7)	22.1 (1.0)	22.2 (1.2)	21.1 (1.5)	0.309

Of note, the number of patients who received a diagnosis of SAT was very similar over the last 5 years. Furthermore, patients who developed SAT in the years before the Sars-CoV-2 pandemic and in 2020 had similar gender and age characteristics. Similarly pre-pandemic and during-pandemic SAT did not differ in terms of the WBC, EAS and CRP levels observed, as well as the thyroid hormonal profile.

## Discussion

The results of the present study show a similar incidence of SAT over the last 5 years. In particular, throughout the 7-month period concomitant to the outbreak of COVID-19 infection, no increase in the diagnosis of SAT was recorded. This observation is corroborated by the fact that the present study was carried out in the Brescia area, the region with the highest prevalence of Sars-CoV-2 infection in Italy [1]. It should be noted that a previous diagnosis of COVID-19 was detected in one patient out of 10 who developed SAT from April 2020 to October 2020.

De Quervain’s thyroiditis shows a typical seasonal variation in its incidence with a higher prevalence in spring and summer [5]. Thus, the present study was specifically designed to take into account the number of SAT diagnoses made in the April–October period of different years.

According to the Rochester Epidemiology Project, SAT is estimated to have an incidence of 12.1 cases per 100,000/year, 5 times higher in women than in men [6], and it is presumed to be caused by a viral infection or a postviral inflammatory process possibly associated to entero, coxsackie or adenovirus [3, 7].

It is interesting to note that these data are very close to what was observed in the Brescia area in recent years. Indeed, in a population of about 200,000 inhabitants we would expect to have 24 cases/year. Thus, the actual number of cases observed in the 7 months of this year as well as in previous years is indeed the number expected in the Brescia area each year.

Brancatella et al. recently described the first case of SAT in a patient affected by Sars-CoV-2 [8]; the authors alerted ‘clinicians to additional and unreported clinical manifestations associated with COVID-19’ on the basis of a chronological association between COVID-19 and SAT disease observed in that patient. Since then several other case reports have been published [9–16].

The pathophysiology of the association between SAT and COVID-19 infection is reported to be similar to the association with other viral conditions targeting the thyroid of genetically susceptible individuals. Muller et al. suggested that this could be due to the presence of angiotensin-converting enzyme 2 (ACE2) receptors, which are more prevalent in thyroid than lung cells [17]. ACE2 receptors are present in different tissues such as myocardium, gastrointestinal mucosa and the respiratory tract [18, 19], all tissues shown to harbour the Sars-CoV-2 virus [20, 21]. More recently, Rotondi et al. demonstrated that the mRNA encoding for the ACE2 receptor is expressed in thyroid follicular cells in human thyroid surgical specimens and in primary cultures of thyroid cells, making them a potential target for Sars-Cov-2 [22]. Based on this evidence, the hypothesis that Sars-Cov-2 could be responsible for SAT appears plausible. However, if COVID-19 would represent a major clinical condition for subsequent SAT development, due for example to a specific tropism of this virus for the thyroid, one would expect an increase in SAT incidence during the COVID-19 outbreak. Conversely, we did not observe any increase in the incidence of SAT during the 7-month period in 2020 as compared to similar periods over recent years. Furthermore, no differences in the male/female ratio nor in the clinical phenotype at presentation were observed in patients who developed SAT in ‘the Sars-Cov-2 year’.

Another and likely more intriguing aspect could be the possibility that COVID-19 patients have taken corticosteroids, thereby masking a contemporary SAT. Unfortunately, it has not been possible to demonstrate or rebut this possibility. In addition, it could be theoretically possible that some patients with SAT were treated by their family physician and did not attend the endocrine outpatient clinic. However, the above possibility should be considered of negligible relevance in view of the fact that from April 2020, remote endocrinological consultation for patients and physicians was established and active and no queries or cases of SAT were received. Thus, although potential limitations might be present, the results reported here appear reliable.

In conclusion, the possibility that COVID-19 could be responsible for the development of SAT, although theoretically possible and documented by several case reports, appears not to be a frequent event. The data reported here do not show an increase of the incidence of SAT in the Brescia area, a region with the highest prevalence of COVID-19 in Italy during the period of the pandemic outbreak.

## Data Availability

The data supporting the findings of this study are available from the corresponding author, upon request.
